# Racial Categorization and Intergroup Relations in Children: The Role of Social Status and Numerical Group Size

**DOI:** 10.3389/fpsyg.2021.719121

**Published:** 2021-10-22

**Authors:** Cassandra Gedeon, Constantina Badea, Rana Esseily

**Affiliations:** ^1^Laboratoire Parisien de Psychologie Sociale, Université Paris Nanterre, Nanterre, France; ^2^Laboratoire Éthologie, Cognition et Développement, Université Paris Nanterre, Nanterre, France

**Keywords:** racial categorization, intergroup relations, social status, numerical group size, children

## Abstract

The aim of this review was to examine the effect of social and numerical group size on racial categorization and intergroup relations in children. We first described the development of racial categorization and the factors that increase the saliency of the race criterion in different contexts. Then, we examine the role of social status in intergroups relations and show that low status children express lower ingroup favoritism compared to their peers from high status groups. Few studies investigated the role of ingroup size on intergroup biases. Here, we look at this numerical variable through the proportion of children of different racial groups in the school environment. The results show that homogeneous environments contribute to the decrease of bias and negative attitudes. We discuss how identifying specific and interactive effects of the social and numerical group size would allow us to implement early and efficient intervention programs.

## Introduction

Social and developmental psychologists have been studying racial issues for many decades (e.g., Aboud, 1988). Even though cultural diversity has been present in our societies for a long time, racial issues have been and are still present in public debates and conflicts still occur in modern societies. The Black Lives Matters movement in the United States typically illustrates how racial issues are at the basis of important social and political conflicts today, despite all efforts of integration policies (Atkins, 2019).

Social categorization is at the basis of intergroup processes that precedes prejudice and discrimination (Bigler and Liben, 2006). In children, racial categorization development depends on social context which includes ingroup size and social status (Verkuyten and Thijs, 2001; Gedeon et al., 2021). These factors modulate children’s experience in a given environment for example in terms of saliency of their group membership and valorization of their social identity and therefore the development of racial categories.

In most studies on intergroup relations, ingroup size and ingroup social status are confounded leading to difficulties in identifying their specific effects. Indeed, often, people who belong to numerically minority groups have also a low social status (economically and in terms of prestige). However, in some contexts, these two factors are not correlated (e.g., White people in South Africa). Do these factors have cumulative negative effects on intergroup relations among children? Is social status more influential than ingroup size when studying prejudice and discrimination?

The aim of this review is to examine the characteristics of the environment in which children grow up (in terms of ingroup size and social status) and their effects on social categorization development and intergroup relations. We will first describe the development of racial categorization and its saliency in some social environments and how it guides social relations. Then we will try to differentiate the specific effects of group size and social status in the literature. For this purpose, we used Psychinfo as an academic search engine and scanned studies published between 2000 and Mid-August 2020. All the studies must have been published in a peer-reviewed journal, included a dependent measure of intergroup bias and used a child sample (mostly preschoolers but all younger than 13 years – preteens). The keywords were: “racial categorization” and “intergroup relations” or “intergroup bias” and “social status” or “numerical group size.” We identified 73 papers and we checked whether the titles and the abstracts fit with our criteria. Finally, we included in the review 17 publications including 26 experimental studies (see Supplementary Table 1).

## The Development of Racial Categorization in Preschool Children

Race awareness is the ability for an individual to recognize race based on physical attributes (Aboud, 1988). This appears very early in a child’s development (Kelly et al., 2005; Hirschfeld, 2008; Hailey and Olson, 2013) and can be considered as a premise of the racial categorization process. Race is a social category based partly on physical characteristics such as skin color, hair color and type, and facial features. Racial categorization can be defined as “the tendency for race to be perceived as a psychologically salient and meaningful basis for grouping others” (Pauker et al., 2016, p. 33).

One of the factors that influence social categorization is the saliency of the criterion used in the categorization process. For example, children are able to categorize others as young or old people if age is salient in a given context (Rhodes and Baron, 2019). According to the self-categorization theory, the saliency of a criterion depends on the ratio between perceived intergroup differences and ingroup similarities (Turner and Reynolds, 2011). Thus, children should be able to compare these perceptual differences in order to use any criteria for grouping people.

Forming social categories in children depends both on their cognitive development and on their social experiences (Gedeon et al., 2021). Indeed, social categories do not develop simultaneously and their emergence depends on the age of the child and his or her social environment. Developmental studies show that under 3 years of age, children seem to have a lack of awareness to race as a meaningful social category whereas gender, age and language for example seem to be better predictors for their attitudes and for guiding their behaviors (for a review, see Esseily et al., 2016).

This late racial categorization development can be explained both on an ontogenetic and phylogenetic level. Indeed, according to an evolutionary perspective, human tendency to encode coalitional alliances was based first on gender and age, and those based on race appeared later with the development of long-distance migrations (Cosmides et al., 2003). One other explanation for the later use of racial criterion in children is that social categorization is guided by familiarity and is therefore highly dependent on the children’s environment (Pauker et al., 2017). For example, children are exposed earlier to gender differences than race because they have been exposed since birth to individuals of both genders (Quintana, 2012). In contrast, their experiences with members of other races are very context-dependent (Kurtz-Costes et al., 2011). Children who live in a highly diversified environment are more familiar with individuals of different racial groups than those who live in low diversified contexts.

In fact, according to the Developmental Intergroup Theory (DIT; Bigler and Liben, 2006, 2007), in order to understand the development of stereotyping and discrimination in children, one must focus on the environmental factors that impact this development. One of the components of the DIT is the psychological salience of a person’s membership that depends on the proportional group size (Bigler and Liben, 2006, 2007). In general, being a member of a numerically minority group in a social context makes the group affiliation more salient compared to being part of a majority group (Badea and Askevis-Leherpeux, 2005). Imagine the situation where a woman is alone in an elevator with men. She is more likely to think of this category of membership (i.e., woman) than in the situation where there are men and women in equal proportions. When the numerical group size of the ingroup is minority, its members are more aware of their group membership, compared to those who have a majority numerical group size.

Most of the studies on social categorization with children do not consider the ingroup characteristics in terms of group size but focus on social status. We will describe in the next section these studies and then we will present the few studies that took into account the group size. As far as we know, the effect of numerical group size is not directly examined in the literature. This is why we will include studies that have considered the role of school diversity in the relationships between children, referring to the numerical group size. In reality, a minority group often has low social status (Sachdev and Bourhis, 1991), but for our proposal we distinguish between social and numerical group size by using the term “minority” to refer only to numerical group size and we will try to disentangle their specific effects.

## The Impact of Social Context on the Perception of Race and Intergroup Relations

### The Effect of Ingroup Social Status

Children belonging to low social status groups tend to be more aware about race compared to children belonging to high social status groups (Akiba et al., 2004). Low social status children categorize others by race more readily (Kinzler and Dautel, 2012), and integrate conceptual knowledge about race earlier compared to high social status children (Ho et al., 2015). For example, Kinzler and Dautel (2012) compared 5-6-year-olds’ reasoning about the stability of race and language throughout an individual’s life. English-speaking children (White and Black) were presented with a series of images of children from both races, paired randomly with either a voice clip in English or in French. Participants were then presented with images of adults that also varied in terms of race and language and children were asked to match each child photo with an adult photo according to how he/she imagines the child will grow up. White children (with high social status) matched the images upon the language criteria, whereas Black children (with low social status) matched the images upon race, suggesting a more stable race-based categorization among children coming from low status environments.

This ability to categorize others by race in low status children develops with age (for a review, see Bonvillain and Huston, 2000; Hailey and Olson, 2013). Indeed, a study with older children (middle-schooled children) showed that members of a low-status racial group show a greater awareness of racial stereotypes and discrimination than children belonging to high social status racial group (Dulin-Keita et al., 2011). Authors examine whether children of low status racial groups have an awareness of race at earlier ages than those from high status groups and whether they experienced racial discrimination. Results showed that Black children were able to define race more accurately, and Hispanic children encountered more racial discrimination, compared to children from high social status group (i.e., White children). These findings may be related to differences in socialization between children with high-status and low-status racial groups. In fact, parents of low-status children are more likely to explain to their children certain distinctions they may observe on the basis of race, at a younger age than parents of the high-status group (for a review, see Priest et al., 2014).

Children from high status groups are also influenced by their socialization in the use of social categories and stereotypes. The social learning approach suggests that stereotypes are learned from the social environment in which children live, based on children’s actual observations of differences between groups (e.g., Eagly et al., 2000). For example, Bar-Tal (1996) examines social categorization in 2.5–6.5 year old children in Israel in an environment of intergroup conflict. Findings indicate that children’s daily environment has a profound effect on their concept formations of social groups. Children as young as 2.5 years-old are able to categorize negatively the “Arabs” using stereotypes that they hear or observe regularly.

Once internalized through socialization, social categorization has consequences for intergroup relations. Racial categorization allows children, as well as adults, to define themselves as members of a group and motivates them to favor their ingroup over the outgroup, in order to maintain a positive image of their group, a rewarding social identity (Tajfel and Turner, 1986). However, members of low-status groups may show an opposite tendency to favor the outgroup (Jost and Burgess, 2000). Preschool children follow a similar pattern as adults regarding intergroup attitudes from high-status and low-status racial groups, where children from high-status groups show robust ingroup favoritism but children from low-status groups show more mixed outcomes (e.g., out-group favoritism, pro-White bias, or neither in-group or out-group favoritism; Corenblum and Annis, 1993; Griffiths and Nesdale, 2006; Gedeon et al., 2021). This expression of an out-group bias by low-status children can be the reflection of an internalization of prejudices and intergroup discrimination in society (Bonvillain and Huston, 2000; Masse et al., 2009). For example, one study shows a tendency to favor the outgroup among 5 to 8 years-old Native Indian children who had more positive attitudes toward the White group than toward their own-group (Corenblum and Annis, 1993).

These studies show that the social status of groups can have an impact on the development of racially based social categorization, as well as on children’s intergroup relationships. Another factor that may have an important role in racial categorization and that was less studied in the literature is the children’s school environment, in which racial groups may be found in different proportions (i.e., ingroup size).

### The Effect of Ingroup Size in School Environments

The diversity of an environment impacts intergroup processes such as categorization, in-group favoritism or the perception of intergroup cultural distance (Pauker et al., 2017). According to the familiarity-based theory, children growing up in multiracial environments learn racial labels and distinctions sooner than those in monoracial settings (Ramsey, 2008). Indeed, studies showed that when children were attending a racial homogeneous or heterogeneous school, findings were completely different. Rutland et al. (2005) showed that 3-to-5-year-old children in racially mixed preschool classrooms did not show evidence of a bias in favor of their White in-group, whereas children in racially homogeneous classrooms did demonstrate bias. These findings were replicated with older children: McGlothlin and Killen (2010) showed that White American 7-to-10-year-old-children demonstrated more ingroup racial bias and were less likely to consider cross-race dyads (Black and White children) as friends when attending homogeneous schools, compared to those attending racially heterogeneous schools.

Homogeneity versus heterogeneity of school environments in these studies refer to the proportion of minority children in the school. In most studies the numerical majority corresponds to the high-status racial group, but the proportions may be reversed in disadvantaged neighborhoods. Thus, a homogeneous environment includes primarily children from the high-status racial group, with very few children from low-status groups represented. A heterogeneous school environment includes children from different racial groups in equivalent proportions. In addition to children’s familiarity with other racial groups, school diversity allows us to examine the impact of group size on intergroup relations.

In a study conducted in France with preschool children (4–6 years old), Gedeon et al. (2021) examined the impact of ingroup size in a low diversified school environment on racial categorization and perceived cultural distance. Authors used a spontaneous social categorization task using pictures of children from different racial groups broadly represented in France (Europeans, Black-, and North-Africans), and an evaluation of the perceived cultural distance between participants’ in-group and the racial group represented in the picture, adapted to children and based on three factors (language, eating habits, and music). Results revealed an effect of age on racial categorization: the older the children, the more successful they are in this task. They showed that members of the majority group perceived photographs representing minority peers as more different than those representing majority peers. In contrast, participants belonging to minority groups perceived no differences between photographs, according to the racial criteria.

Being numerically in the minority relative to another group makes the distinction between ingroup and outgroup more important for children from the minority group (Brewer et al., 1993; Fishbein, 1996; Brewer and Brown, 1998). Just like the impact of social status, majority children tend to show ingroup favoritism, while children from minority groups tend to show less ingroup bias (Aboud, 1988). This tendency seems to emerge very early in infancy. Indeed, the study by Pun et al. (2016) showed that by 6 months of age, infants were able to infer social dominance between two groups using numerical group size cues, suggesting an evolutionary pattern of intergroup relationships and social dominance. In this study, authors presented infants between the ages of 6 and 9 month with short animations that depicted the actions of two individuals (cartoons) belonging to two groups that differed in numerical size. Infants were first familiarized with the cartoons and then, they saw both individuals bumping into each other. Infants’ looking time to each trial was recorded. Authors reasoned that if infants use numerical group size to infer which cartoon is more dominant, then infants should be more surprised and therefore look longer when the individual from the numerically smaller group prevails. The results confirmed these expectations and this was the only study that investigated the numerical group size *per se*.

### Specific Versus Interactive Effects of Social Status and Ingroup Size

We have presented work that separately examined the role of ingroup social status and the role of ingroup size in racial social categorization and intergroup relations. However, in Western societies, the size of the group and its social status are often confounded: a numerically majority group also has a high social status and conversely, a numerically minority group often has a low social status. Illustrating this confound, studies show that children use race and numerical group size to predict social status. For example, a study with 3.5-to-7-year-old children showed that as young as 3.5 years-old children were able to predict wealth (i.e., who lived in a fancier house, linked to social status) using race. In addition, results showed that this prediction was stable across age (Mandalaywala et al., 2020). However, from a theoretical perspective, it is important to understand whether these factors have cumulative effects or interact in a different manner, especially in early stages of children’s interactions in order to define specific and efficient educational and preventive interventions.

Brown and Bigler (2002) examined the effects of relative group size and social status on the development of children’s intergroup attitudes. Elementary school children (5–11 years old) attending a summer school program were assigned to larger (i.e., majority) or smaller (i.e., minority) novel groups in their classroom (denoted by colored tee-shirts). In addition, the classroom environment contained implicit messages about group status. Large posters conveyed information about traits associated with group membership (spelling ability, leadership ability, athletic prowess, classroom behavior, or occupational prestige). However, in this study the two factors (size and status) were not crossed. High status was associated with the majority group, and low status was associated with the minority group. Children’s intergroup attitudes (e.g., trait ratings, group evaluations) were assessed following several weeks in the classroom. Results showed that, when children are included in a minority group, they show more biased trait ratings (i.e., characterized the outgroup with more negative traits) than majority children, independently of age. When adding the social status, results showed that younger children expressed higher ingroup favoritism than the high-status majority peers. But this result was not found with older children: low-status minority groups expressed lower ingroup favoritism compared to higher-status majority children. These findings suggest that group size may play an important role in the expression of ingroup favoritism and the maintenance of this phenomenon through age. Social status, on the other hand, can sometimes reduce this favoritism and even reverse it in favor of outgroup favoritism, which contributes to the maintenance of inequalities.

An important remark is that studies included in this theoretical note were mostly conducted in WEIRD-populations where often White people are in majority and have high social status (see Supplementary Table 1). It would be interesting to investigate interaction between group size and social status in non-WEIRD-populations where the overlap between the two factors can be less important.

## Conclusion

In this review, we focused on the interactive and the specific effects of group size and social status when studying the development of intergroup processes in children. Findings show that racial categorization appears earlier in low status and minority children compared to high status and majority children. One of the mechanisms that can explain these results is the fact that children socially learn prejudices and discrimination toward their own group (Priest et al., 2014). Unlike their high status peers, low status children face a disparity between the positive attitudes associated with the development of their own group identity and the awareness of the perceived negative value of that identity in the society (Corenblum and Annis, 1993). This is expressed in the absence of a robust ingroup favoritism or even in an outgroup positive bias, contributing in maintaining prejudice and discrimination but also a social hierarchy in general.

Another finding of that review is that social and numerical group size are generally confounded in the literature. Indeed, in most of the studies mentioned here, low status children are also numerically in minority in their school environment. Even though we tried to separate them here in order to examine their specific effect, it is important to bear in mind that this distinction remains hypothetical because in real life they overlap most of the time. However, future studies should aim at identifying the specific effects of social status and ingroup size as well as their interactive effect. This approach would allow early and efficient intervention programs that focus either on the social status on a general level (e.g., Nasie et al., 2021) or on the proportion of racial groups included in school environments (e.g., Verkuyten and Kinket, 2000; Gaias et al., 2018).

## Author Contributions

CG: did the bibliographic research and wrote the first version of the manuscript. CB: revised the manuscript and restructured some parts. RE: revised the final version of the manuscript and made some amendments. All authors contributed to the article and approved the submitted version.

## Conflict of Interest

The authors declare that the research was conducted in the absence of any commercial or financial relationships that could be construed as a potential conflict of interest.

## Publisher’s Note

All claims expressed in this article are solely those of the authors and do not necessarily represent those of their affiliated organizations, or those of the publisher, the editors and the reviewers. Any product that may be evaluated in this article, or claim that may be made by its manufacturer, is not guaranteed or endorsed by the publisher.
