# Supplementary material for: Racial Categorization and Intergroup Relations in Children: The Role of Social Status and Numerical Group Size
Source: Front Psychol. 2021 Oct 22;12:719121. doi: 10.3389/fpsyg.2021.719121 (PMC8568878; doi:10.3389/fpsyg.2021.719121)
Supplement: Supplementary file 1 [file Table_1.DOCX]

**TABLE 1.**

*Description of geographical location, sample size, participants’ age and ethnicity, methods and intergroup bias of included studies.*

| N° | Article | Geographical location of the study | Sample size | Participants’ age range | Participants’ ethnicity | Social status | Numerical group size | Intergroup bias studied | Main results |
| --- | --- | --- | --- | --- | --- | --- | --- | --- | --- |
| 1 | Bar-Tal, 1996 | Israel | *N* = 40 | 2.5-3.5-year-olds and 5.5-6.5-year-olds | Israeli children | / | / | Acquisition of the concept “an Arab” and the development of stereotypes by Israeli children | In an environment of intergroup conflict, children developed negative biases at a younger age than when there is no conflict  Indeed, as early as 2.5 years of age, children had a conception of "Arabs" as bad and expressed stereotypes against this social group |
| 2 | Brown & Bigler, 2002 |  |  |  |  |  |  |  |  |
|  | Study 1 | USA | *N* = 95 | 5-to-11-year-old-children | 90 Euro-Americans, 3 Asian Americans, 2 African Americans | / | Children were assigned to majority, minority or equally distributed groups using a minimal group paradigm | Trait stereotyping, Group evaluations, Peer preferences | No effects of relative group size  All children rated their ingroup more positively than the outgroups |
|  | Study 2 | USA | *N* = 60 | 5-to-10-year-old-children | 59 Euro-American and 1 African American | / | Replication of the first study procedure with smaller minority groups | Trait stereotyping, Group evaluations, Peer preferences,  Perception of similarity to groups | Minority children nominated the other minority child in their classroom as a best friend although more minority children reported that they would rather belong to the majority group |
|  | Study 3 | USA | *N* = 74 | 5-to-11-year-old-children | 71 Euro-American and 3 Asian-American | The social status of the group was added on posters in the classroom | Here, the minority groups were associated with low status traits while majority groups were associated with high status traits | Trait stereotyping,  Group evaluations,  Perception of groups’ stereotypes  Affect about posters | Only older children belonging to low status minority groups showed lower levels of ingroup bias than those belonging to high status majority groups  And these results were found only when the measure was focused on group traits but not individuals traits |
| 3 | Corenblum & Annis, 1993 | Canada | *N* = 294 | 3-to-8-year-old-children | 203 White children  91 Indian children | In this study, Indian children represented the minority group in terms of status and numerical group size. Those factors are confounded here | | Through photographs of Black, White and Indian children, children were asked questions about racial recognition, racial identity, racial preference and social distance  And then six weeks later, children completed a measure of concrete operational thinking and a self-esteem task | Indian children (i.e., the minority group in terms of social status and numerical group size) had more positive attitudes towards Whites than towards their ingroup members Whereas White children chose pictures of their ingroup members when answering positively and outgroup members where answering negatively. Indian children showed conflicted emotions about being a member of a minority group |
| 4 | Gaias et al., 2018 | USA | *N* = 670 | Longitudinal study : at 1 month-old, 54 months-old, first grade and third grade | 80% White, 10% African American, 5% Latino  5% other races or ethnicities | A dichotomous indicator of the children’s minority status (0 = Non-White, 1 = White) was created. This factor was only considered as a covariate in the analyses | The numerical group size was taken into account through the school level diversity and the classroom level diversity | Cross-race friendship  Racial bias: Ethnic Preference and Identity Measure | Cross-race friendship predicts decreased racial bias. Exposure to diversity in the preschool had longitudinal implications on racial bias and cross-race friendship across childhood, these classrooms can promote long-lasting intergroup relationships |
| 5 | Gedeon, Esseily & Badea, 2021 | France | *N* = 87 | 4-to-6-year-old children | 47 ethnic-majority members  40 ethnic-minorities members | / | The numerical group size of each was taking into account through the school level diversity | Children completed a free categorization task using photographs of 3 racial groups (Caucasian, Black- and North-African) and a measure of intercultural distance | Racial categorization increased with age but did not differ across groups. Majority children perceived high intercultural distance while minority children did not show this bias |
| 6 | Griffiths & Nesdale, 2006 | Australia | *N* = 119 | 5-to-12-year-old children | 59 Anglo-Australian and 60 Pacific Islander | They used the social status factor through participant’s ethnic groups | / | Explicit attitudes by using an adapted measure of the Preschool Racial Attitude Measure II.  Implicit attitudes by using the street exercise measure | In terms of implicit and explicit attitude measures, majority children rated their ingroup more positively and showed an ingroup favoritism compared to minority children who rated ingroup and outgroups equally |
| 7 | Kelly et al., 2005 |  |  |  |  |  |  |  |  |
|  | Study 1 | UK | *N* = 64 | 16-to-120-hours newborns | Caucasian | / | / | Visual-Preference task through 32 photographs of adult faces from four ethnic groups (Caucasian, Middle Eastern, Asian and African) | At birth, newborns showed no preferences for faces from ingroup or outgroups |
|  | Study 2 | UK | *N* = 64 | 3-month-old infants | Caucasian | / | / | Identical to study 1 | Infants showed a visual preference for own-race faces |
| 8 | Kinzler & Dautel, 2012 |  |  |  |  |  |  |  |  |
|  | Study 1 | USA | *N* = 16 | 5-to-6-year-old children | 81% White,  One child was White/Asian, one child was Asian and one child’s parent did not report | / | Experimenters tested in a racially heterogeneous environment | Children were presented with a series of trials where they saw an image of a child who was either White or Black and spoke in English or French. After they were shown two adults: one matching the target child’s language but not race and the other one matching the target child’s race but not language. Children were supposed to tell which adult the target child grew up to be | When children had to choose between race and language, they chose the language-match over the race-match as being more stable across an individual’s lifespan |
|  | Study 2 | USA | *N* = 24 | 5-to-6-year-old children | 100% White | / | Experimenters tested in a racially homogeneous environment in order to observe the influence of the context | Identical to the 1^st^ study | Same results than in Study 1 but in a racially homogeneous environment |
|  | Study 3 | USA | *N* = 16 | 9-to-10-year-old children | 100% White | / | Experimenters tested in an racially heterogenous environment | Identical to Study 1 and Study 2 | Children chose the race-match more often than the language-match |
|  | Study 4 | USA | *N* = 24 | 5-to-6-year-old children | 96% African American  One child African American/other | They used the same procedure with African American children –  a minority racial group – in order to make a comparison with the majority group (Study 1 and 2) | Experimenters tested in an racially heterogenous environment | Identical to Study 1, 2 and 3 | 5-to-6-year- old- African Americans chose the race-match more often than the language-match  Because of their different experiences with race as a meaningful social category, race is more stable than language |
| 9 | Kurtz-Costes et al., 2011 | USA | *N* = 108 | 3-and-5-year-old girls (60 3-year-olds and 48 5 year-olds) | 46 Black  62 White | Social status was implied through racial group membership (White or Black) | / | They used a dolls’ birthday party scenario using 4 dolls – one White boy, one White girl, one Black boy and one Black girl – and they scored preferential behavior | Children showed preferential behavior linked to gender and race.  The same-race girl doll favoritism was more pronounced among older children (5 year old) than younger ones.  Also when asked “what doll do you like most?”44% of the 3-year-old Black children selected the White matched gender doll |
| 10 | Mandalaywala et al., 2020 |  |  |  |  |  |  |  |  |
|  | Study 1 | USA | *N* = 215 | 3.5-to-6.9-year-old children | 33% White, 13% Hispanic, 16.7% Asian, 10.7% Black  14.9% Multiracial  11.7% other or not provided | / | / | The rope task provided a measure of children’s tendencies to use gender as a cue to social status, defined in terms of social power and wealth. Wealth-matching task and Social preferences | Young children used gender to predict the status of others. Male participants rated boys as having a higher status than girls across all ages and measures. While female participants rated boys and girls equally.  Also the more the female participants were older, the less they rated girls as having a high social status |
|  | Study 2 | USA | *N* = 205 | 3.5-to-6.9-year-old children | 34.6% White 10.7% Hispanic 12.2% Asian 12.7% Black 14.6% Multiracial  15.1% other or not provided | Race and social status are confounded |  | The procedure was identical to the first study but the rope task was used as a measure of children’s tendency to use race as a cue to social status. The stimuli presented a Black-White racial contrast | Children did not use race to predict status when status included information about social power but did when status was defined by wealth. For example, they used race to predict who lived in a fancier house |
| 11 | Masse et al., 2009 | France | *N* = 320 | 9-to-11-year-old children | 160 Caucasian  160 Black-African | Race and social status are confounded : Caucasian children have a high-social status and Black-African have a low- social status | / | Language analysis based on a story including Black or White characters acting in a positive or a negative manner (race and action were crossed) | No differences between the Caucasian and the Black-African groups: when the action was positive, both groups showed ingroup favoritism while when the action was negative, they showed outgroup favoritism |
| 12 | McGlothlin & Killen, 2010 | USA | *N* = 302 | 7-and-10-year-old children | 94 White-American in a heterogeneous school,  70 Black-American in a heterogeneous school and 138 White-American in a homogeneous school | Race and social status are confounded : White-American have a high-status and Black-American have low-status | Two types of schools : ethnically homogeneous (with >85% of White-American) and ethnically heterogeneous (with <65% of White-American) | Ambiguous Situations Task : moral transgressions made either by a White or a Black character.  Intergroup Contact (only in the homogeneous sample) | White-American children in homogeneous schools demonstrated an ingroup racial bias when evaluating peer situations and were less likely to put cross-race dyads as friends than White-Americans and Black-Americans in heterogeneous schools |
| 13 | Nasie & Diesendruck, 2021 | Israel | *N* = 82 | 3-to-4-year-oldand 5-to-6-year-old | Israeli children | / | / | They used pictures of three racial groups children : a Jew (representing the ingroup), an Arab (representing a conflict outgroup) and a Scot (representing a neutral outgroup). After seeing the pictures, the children were asked what they like to know about them and the experimenter responded either entirely, halfway or not at all | Children asked the most questions about an Arab, then about a Scot and the least about a Jew. The content of their questions revealed four types of properties: psychological characteristics, appearance, personal identity and social identity.  Older children (5-to-6-year olds) always showed more positive attitudes towards ingroup members than outgroups. 3-to-4-year olds showed more positive attitudes towards in- and out-groups when given a complete answer to their questions, compared to when they received partial or no answers |
| 14 | Pun et al., 2016 | Canada |  |  |  |  |  |  |  |
|  | Study 1 |  | N = 48 | 9-to-12-month-old infants | 47% Caucasian  32% East Asian  21% other ethnicities | / | / | Experimenters used short animations that showed actions and goals of two groups of agents, each group differing in numerical size and color. Infants’ looking time was measured in each of the trials showing conflictual and dominance relationships between the two groups | Infants looked longer when an agent belonging to the majority group surrendered to the minority agent, compared to the expected outcome namely where the majority agent didn't surrender to the minority agent |
|  | Study 2 |  | N = 48 | 6-to-9-month-old infants | 66% Caucasian  18% East Asian  16% other ethnicities | / | / | The procedure was the same as the 1^st^ study | As in study 1, infants used the relative numerical size of two groups to infer the social dominance relationship: they looked longer when the agent belonging to the minority group was dominant compared to the opposite situation |
|  | Study 3 |  | *N* = 48 | 6-to-12-month-old infants | 41% Caucasian  24% East Asian  35% other ethnicities | / | / | In this study, the conflictual situation between both groups (minority and majority) is eliminated. The agents are attempting to cross a barrier (a non-conflicting goal) | Infants did not look longer at either group showing that the looking times were not driven only by the numerical group membership of the agents |
| 15 | Rutland et al., 2005 | UK | *N* = 136 | 3-to-5-year-old children | Anglo-British | / | Three types of environments : (a) majority White preschool (with no minorities), (b) majority White preschool (with 10% of racial minority children) and (c) racially mixed preschools (with at least 50% of racial minority children) | Racial intergroup bias, interracial contact, racial consistency and stereotype trait were measured using photographs of children and adults coming from four ethnic groups : Anglo-British, African-Caribbean, Asian-Indian and Far-East Indian | Children in the all-White and majority-White environments showed ingroup favoritism on positive and negative trait attribution whereas this was not found in children in racially mixed preschools. Anglo-British children in heterogeneous schools were less positive about their ingroup and less negative about the outgroup |
| 16 | Verkuyten & Kinket, 2000 | Netherlands | *N* = 231 | 10-to-12-year-old children | Dutch | / | Homogeneous and heterogeneous classroom environments | Contact, social distance and prejudice were measured towards five ethnic outgroups : Indonesians, Moroccans, Surinamese, Turks and Yugoslavs | Dutch children formed an ethnic hierarchy among ethnic groups as follows: Dutch, Indonesians, Surinamese, Yugoslavs, Moroccans and then Turks. The lower an ethnic group is in this hierarchy, the more they’re prejudiced and the more social distance towards them observed. In addition, social distance depended on the context: less social distance was found towards ethnic minority groups in racially heterogeneous classrooms compared to homogeneous classrooms |
| 17 | Verkuyten & Thijs, 2001 | Netherlands | *N* = 667 | 10-to-13-year-old children | 557 Dutch  110 Turkish | Race and social status are confounded :Dutch children have a high-social status and Turkish children have a low-social status | Homogeneous and heterogeneous classroom environments | Gender and ethnic identification, intergroup bias, ethnic bias and gender bias | Turkish children had higher ethnic identification compared to Dutch children. But the ethnic bias and ingroup favoritism was stronger among Dutch children than Turkish children |
